# Sequence of clinical and neurodegeneration events in Parkinson’s disease progression

**DOI:** 10.1093/brain/awaa461

**Published:** 2021-02-05

**Authors:** Neil P Oxtoby, Louise-Ann Leyland, Leon M Aksman, George E C Thomas, Emma L Bunting, Peter A Wijeratne, Alexandra L Young, Angelika Zarkali, Manuela M X Tan, Fion D Bremner, Pearse A Keane, Huw R Morris, Anette E Schrag, Daniel C Alexander, Rimona S Weil

**Affiliations:** 1 Centre for Medical Image Computing, Department of Computer Science and Department of Medical Physics and Biomedical Engineering, UCL, London, UK; 2 Dementia Research Centre, UCL Institute of Neurology, UCL, London, UK; 3 Department of Neuroimaging, Institute of Psychiatry, Psychology and Neuroscience, King’s College London, London, UK; 4 Department of Clinical and Movement Neuroscience, UCL Queen Square Institute of Neurology, UCL, London, UK; 5 Movement Disorders Consortium, UCL, London, UK; 6 Neuro-ophthalmology, National Hospital for Neurology and Neurosurgery, University College London Hospitals, London, UK; 7 Institute of Ophthalmology, UCL, London, UK; 8 Moorfields Eye Hospital, London, UK; 9 The Wellcome Centre for Human Neuroimaging, UCL Institute of Neurology, UCL, London, UK

**Keywords:** event-based model, disease progression, Parkinson’s disease, dementia, vision

## Abstract

Dementia is one of the most debilitating aspects of Parkinson’s disease. There are no validated biomarkers that can track Parkinson’s disease progression, nor accurately identify patients who will develop dementia and when. Understanding the sequence of observable changes in Parkinson’s disease in people at elevated risk for developing dementia could provide an integrated biomarker for identifying and managing individuals who will develop Parkinson’s dementia. We aimed to estimate the sequence of clinical and neurodegeneration events, and variability in this sequence, using data-driven statistical modelling in two separate Parkinson’s cohorts, focusing on patients at elevated risk for dementia due to their age at symptom onset. We updated a novel version of an event-based model that has only recently been extended to cope naturally with clinical data, enabling its application in Parkinson’s disease for the first time. The observational cohorts included healthy control subjects and patients with Parkinson’s disease, of whom those diagnosed at age 65 or older were classified as having high risk of dementia. The model estimates that Parkinson’s progression in patients at elevated risk for dementia starts with classic prodromal features of Parkinson’s disease (olfaction, sleep), followed by early deficits in visual cognition and increased brain iron content, followed later by a less certain ordering of neurodegeneration in the substantia nigra and cortex, neuropsychological cognitive deficits, retinal thinning in dopamine layers, and further deficits in visual cognition. Importantly, we also characterize variation in the sequence. We found consistent, cross-validated results within cohorts, and agreement between cohorts on the subset of features available in both cohorts. Our sequencing results add powerful support to the increasing body of evidence suggesting that visual processing specifically is affected early in patients with Parkinson’s disease at elevated risk of dementia. This opens a route to earlier and more precise detection, as well as a more detailed understanding of the pathological mechanisms underpinning Parkinson’s dementia.

## Teaser

Using event-based modelling, Oxtoby *et al*. reveal the fine-grained probabilistic sequence of changes in clinical, cognitive and neuroimaging measures during Parkinson’s disease progression. Performance on visual tasks is impaired early on, typically before neurodegeneration and retinal thinning can be detected.

See Le Heron *et al*. (doi:10.1093/brain/awab060) for a scientific commentary on this article.

## Introduction

Dementia is one of the most debilitating aspects of Parkinson’s disease, with important social and economic implications (Spottke *et al.*, 2005; Leroi *et al.*, 2012). It affects half of all patients within 10 years of diagnosis but with high variability in the timing of onset (Williams-Gray *et al.*, 2013). There are no validated biomarkers of Parkinson’s disease progression, and the particular sequence and evolution of Parkinson’s disease pathology and cognitive decline remain unclear. Knowing the precise sequence of events in Parkinson’s disease progression will be critical for: (i) reducing heterogeneity in clinical trials; (ii) monitoring treatment outcomes as new therapeutic interventions are developed; and (iii) providing important insights into the mechanistic underpinnings of degeneration in Parkinson’s disease. Therefore, a key challenge is to construct quantitative models of pathological and cognitive decline in Parkinson’s disease progression using real-world patient data such as neuroimaging and clinical measures.

Pathological changes underlying the development of dementia in Parkinson’s disease relate to accumulation of α-synuclein (Spillantini *et al.*, 1997), as well as amyloid-β, and tau (Irwin *et al.*, 2017) with a synergistic relationship seen between these proteins (Compta *et al.*, 2011; Swirski *et al.*, 2014). However, it is challenging to detect these processes in patients living with the disease. There is no radio-ligand that directly binds α-synuclein. Even compounds that directly bind to amyloid, such as Pittsburgh compound B, have low specificity for predicting dementia in Parkinson’s disease (Gomperts *et al.*, 2012). The utility of tau-binding ligands has been studied longitudinally in Alzheimer’s disease research (Jack Jr *et al.*, 2018), but they are yet to be tested in Parkinson’s disease, and may be similarly afflicted by low specificity. More generally, molecular neuroimaging markers are costly and not widely available.

Conventional neuroimaging features that measure tissue loss caused by neuronal death, such as cortical thickness, are less likely to be sensitive to early stages of Parkinson’s dementia (Hattori *et al.*, 2012). More advanced techniques have begun to show potential to identify tissue changes related to neurodegeneration (Lanskey *et al.*, 2018). Of these, quantitative susceptibility mapping (QSM) is particularly promising as it relates to accumulation of brain tissue iron, which is strongly linked with neurodegeneration (Ward *et al.*, 2014; Ndayisaba *et al.*, 2019), co-localizes with amyloid and tau (Ayton *et al.*, 2017), and correlates with cognitive change in Parkinson’s disease (Thomas *et al.*, 2020).

Neuropsychological tests across cognitive domains are used to map cognitive decline in Parkinson’s disease. Tests of verbal fluency and visuospatial function seem to be early indicators of an individual’s risk of developing dementia (Williams-Gray *et al.*, 2013), and visual processing regions are affected early in patients who develop cognitive impairment (Weil *et al.*, 2016). Indeed, multiple lines of evidence support the importance of visual processing regions in Parkinson’s dementia and cognitive decline. These include accumulation of post-mortem pathology (Toledo *et al.*, 2016), deficits in colour vision (Anang *et al.*, 2014), and our own studies highlighting deficits in quantitative higher-order visual measures (Weil *et al.*, 2019) and retinal thinning (Leyland *et al.*, 2020).

How these clinical and neuroimaging measures of decline fit into a sequential model of progression to Parkinson’s dementia is not yet known. A particular challenge is the absence of any objective pathological biomarkers that track Parkinson’s disease progression in patients at elevated risk of dementia—unlike Alzheimer’s disease, for example, where neuroimaging and CSF biomarkers are now included in diagnostic criteria (Dubois *et al.*, 2014). Disease duration (time since diagnosis), which can be useful as a measure of disease progression in other degenerative diseases such as Alzheimer’s disease, is particularly ineffective in Parkinson’s dementia (Prange *et al.*, 2019). Duration is often negatively related to disease progression as patients with an older age at diagnosis frequently progress more rapidly to dementia while having a shorter duration of disease (Marinus *et al.*, 2018; Prange *et al.*, 2019). Another approach to consider in modelling progression to Parkinson’s dementia is regressing measures of interest against a clinical cognitive variable such as the Montreal Cognitive Assessment (MoCA) (Nasreddine *et al.*, 2005). However, this clinical measure is confounded by practice/learning effects (in general) and by ceiling/floor effects at early/late stages of cognitive involvement. Data-driven approaches that jointly estimate disease progression and the unknown (latent) disease stage are an emerging approach (Iddi *et al.*, 2018; Li *et al.*, 2019). In the work of Iddi and colleagues (2018) this approach was applied to estimate disease progression in a general Parkinson’s population using mostly clinical features.

A data-driven understanding of Parkinson’s disease progression in patients at elevated risk of dementia will enable robust identification of at-risk patients before dementia has taken hold. Currently the state of the art in Parkinson’s dementia risk determination are clinico-genetic algorithms (Liu *et al.*, 2017; Schrag *et al.*, 2017). These calculate an individual’s risk of cognitive decline by combining demographic, clinical, motor, and cognitive scores, as well as genetic and biomarker results. Across all studies, higher age at onset is consistently and significantly seen as the strongest risk factor for dementia in Parkinson’s disease (Hietanen and Teräväinen, 1988; Jankovic *et al.*, 1990; Biggins *et al.*, 1992; Aarsland *et al.*, 2001, 2007; Akinyemi *et al.*, 2008; Williams-Gray *et al.*, 2013; Liu *et al.*, 2017).

Here we used novel event-based disease progression modelling to investigate Parkinson’s disease progression, enriching for those at higher risk of dementia using older age at onset. The event-based model (Fonteijn *et al.*, 2012; Young *et al.*, 2014) is a generative statistical model of disease progression that learns the ordering (and uncertainty in this ordering) of observable abnormalities from a cross-sectional dataset, without the need for *a priori*-defined disease stages or normal/abnormal cut-points—making it particularly suitable, in principle, for Parkinson’s disease where such disease stages do not exist in a meaningful way. A recent development uses kernel density estimation (Firth *et al.*, 2020) to allow the approach to cope naturally with the ceiling and floor effects seen in clinical data, enabling the extension to Parkinson’s disease for the first time. The model learns directly from biomarker data, where we use the term ‘biomarker’ to include any observable dynamic measure that potentially contains disease information, e.g. clinical/cognitive tests, brain imaging, etc. Previous versions of the event-based model have been used widely in recent years to construct data-driven models of disease progression in sporadic Alzheimer’s disease (Young *et al.*, 2014; Oxtoby *et al.*, 2017), familial Alzheimer’s disease (Fonteijn *et al.*, 2012; Oxtoby *et al.*, 2018), Huntington’s disease (Fonteijn *et al.*, 2012; Wijeratne *et al.*, 2018), and others (Eshaghi *et al.*, 2018; Firth *et al.*, 2018). We update the method of Firth *et al.* (2020) and apply it to the complex and heterogeneous problem of Parkinson’s disease progression.

## Materials and methods

### Participants

We analysed baseline visit data from two separate cohorts. We refer to our local cohort as the discovery cohort, which is from the Vision In Parkinson’s Disease study (Leyland *et al.*, 2020). The second cohort is from the Parkinson’s Progression Marker’s Initiative (PPMI) (Marek *et al.*, 2011). See Table 1 for demographics and statistical comparisons and Supplementary material for additional details.

**Table 1:** Descriptive statistics of study participants

	Controls	PDD-LR	PDD-HR	*U* or χ^2^ (PDD-HR versus Controls)	*P*
					
**Discovery cohort (local study)**	***n = *33**	***n = *64**	***n = *36**
Age, years	64.7 (9.0)	59.7 (5.1)	73.0 (4.0)	266.5	<0.0001
Disease duration, years	–	4.7 (2.6)	3.4 (2.1)	–	–
Age at onset PD	–	55.5 (4.3)	70.1 (3.8)	–	–
UPDRS total	8.2 (5.2)	46.8 (24.0)	42.9 (18.1)	4.5	<0.0001
LEDD	–	484 (284)	388 (201)	–	–
Gender, female/male	18/15	35/29	13/23	1.68	0.195
RBDSQ	1.7 (1.3)	4.2 (2.5)	4.2 (2.4)	218	<0.0001
Smell test (Sniffin’ sticks)	12.2 (2.6)	8.1 (2.9)	6.8 (3.4)	133	<0.0001
Cognition (MoCA)	28.6 (1.3)	28.2 (1.7)	27.6 (2.2)	416.5	0.015
Category Fluency (animals)	22.1 (5.2)	22.0 (5.1)	20.2 (6.4)	459	0.053
Letter Fluency	16.8 (5.6)	16.5 (4.9)	16.3 (6.4)	553.5	0.315
**External cohort (PPMI study)**	***n = *127**	***n = *206**	***n = *146**		
Age	60.0 (11.0)	55.6 (7.3)	70.9 (3.7)	3119	<0.0001
Disease duration, years	–	0.53 (0.55)	0.65 (0.62)	–	–
Age at onset PD	–	55.0 (7.3)	70.3 (3.7)	–	–
UPDRS total	1.8 (2.8)	26.0 (11.1)	30.2 (11.1)	36	<0.0001
Gender female/male	48/79	72/134	48/98	0.52	0.470
RBDSQ	1.9 (1.4)	3.9 (2.5)	4.2 (2.6)	4408	<0.001
Smell test (Sniffin’ sticks equivalent)	13.6 (1.7)	9.3 (3.4)	7.4 (3.6)	1208	<0.0001
Cognition (MoCA)	28.2 (1.1)	27.4 (2.2)	26.8 (2.2)	5409	<0.0001
Category Fluency (animals)	22.0 (5.4)	21.8 (5.5)	19.5 (4.6)	6732	<0.0001
Letter Fluency	14.5 (4.3)	13.2 (4.9)	12.2 (4.4)	6194	<0.0001

Values are mean (SD), except where indicated otherwise. Each *P*-value shown is for a Mann-Whitney U-test (means) or χ^2^-test (proportions) of the null hypothesis that there is no statistical difference between the PDD-HR and control samples. For comparison of olfactory performance, PPMI UPSIT scores were converted to Sniffin’ Sticks equivalent using an equi-percentile method (Lawton *et al*., 2016). LEDD = Levodopa equivalent daily dose; PD = Parkinson’s disease; RBDSQ = REM Sleep Behaviour Disorder Screening Questionnaire; UPDRS = Unified PD Rating Scale; UPSIT = University of Pennsylvania Smell Identification Test.

Our discovery cohort included data from 107 patients with Parkinson’s disease (disease duration 4.1 ± 2.5 years) who were recruited to our UK centre, 37 of whom were diagnosed at age 65 or later, plus 34 healthy control subjects. Throughout we will refer to Parkinson’s patients with age at onset ≥65 as PDD-HR (Parkinson’s Disease Dementia–High Risk), and other patients as PDD-LR (Parkinson’s Disease Dementia–Low Risk). This cohort has been described previously (Leyland *et al.*, 2020). Inclusion criteria were early stage Parkinson’s disease (UK Parkinson’s Disease Society Brain Bank diagnostic criteria) (Gibb and Lees, 1989), within 10 years of diagnosis, aged 49–80 years. Exclusion criteria were confounding neurological or psychiatric disorders (four excluded), a diagnosis of dementia or Mini-Mental State Examination (MMSE) score ≤25 (two excluded) (Liu *et al.*, 2017), or ophthalmic disease sufficient to impair visual acuity (three excluded) (Leyland *et al.*, 2020). Data from 34 unaffected age-matched control subjects, recruited from unaffected spouses and university databases, were also included. All participants gave written informed consent and the study was approved by the Queen Square Research Ethics Committee.

We downloaded PPMI data in April 2020 from the Laboratory Of NeuroImaging portal accessed via the PPMI website (http://www.ppmi-info.org/). PPMI is an observational multicentre study involving over 400 newly diagnosed drug-naive patients (451 downloaded) and ∼200 healthy controls (196 downloaded), plus a small number of possible prodromal individuals [hyposmia or rapid eye movement (REM) sleep behaviour disorder (RBD)], and patients having a PET/SPECT scan without evidence of dopaminergic deficiency (SWEDD), all with standardized clinical, cognitive and neuroimaging assessments. Inclusion criteria for our study was PPMI participants having complete baseline data in the measures of interest (described below) with known disease duration (*n*** **=** **46 excluded). Prodromal and SWEDD groups were omitted from model fitting, and controls with RBD were excluded [REM Sleep Behaviour Disorder Screening Questionnaire (RBDSQ) ≥5] (Stiasny-Kolster *et al.*, 2007). Participants from the PPMI dataset each provided written informed consent at their participating site for their data to be collected and shared for this initiative. Our discovery cohort included a richer set of measures than the PPMI cohort. Our comparison experiments included only those clinical and neuroimaging measures available in both cohorts.

### Clinical and neuropsychological evaluation

Symptom severity was assessed using the Movement Disorders Society Unified Parkinson’s Disease Rating Scale (UPDRS) (Movement Disorder Society Task Force on Rating Scales for Parkinson's Disease, 2003). Participants were tested on their usual medications and levodopa equivalent daily dose (LEDD) was calculated (Tomlinson *et al.*, 2010). Cognition was tested using the MoCA. Olfaction was assessed using Sniffin’ sticks (Hummel *et al.*, 1997) in the discovery cohort, and the University of Pennsylvania Smell Identification Test (UPSIT) in the PPMI cohort. Participants also completed the Hospital Anxiety and Depression Scale (HADS; Snaith, 2003), and RBDSQ (Stiasny-Kolster *et al.*, 2007). Control patients scoring ≥5 were excluded (discovery cohort *n*** **=** **1; PPMI cohort *n*** **=** **34), as these could be considered REM sleep behaviour disorder cases rather than true unaffected controls (Stiasny-Kolster *et al.*, 2007). Cognitive assessment was in line with recent MDS guidelines (Litvan *et al.*, 2012) with two assessments per cognitive domain, as described previously (Leyland *et al.*, 2020) (Table 1).

### Assessments of visual function

Visual acuity was measured using a LOGMAR chart and contrast sensitivity was measured using a Pelli-Robson chart (SSV-281-PC) (http://www.sussex-vision.co.uk). Colour vision was assessed using the D15 test (Farnsworth, 1943) and error scores log transformed. Higher-order visuo-perception was measured using two contrasting tasks, that each probe distinct aspects of higher-order visuo-perception: the Cats-and-Dogs test that measures skew tolerance (Weil *et al.*, 2017; Leyland *et al.*, 2020) and biological motion (Saygin, 2007). These tests were administered at the start of each testing session using a counterbalanced design to control for order effects. Stimuli were generated within MATLAB Psychophysics Toolbox 3 and implemented on a Dell Latitude 3340, in a darkened room.

### Ophthalmic assessments and retinal structure

A comprehensive ophthalmic assessment was performed by a consultant ophthalmologist (F.B.) and included slit-lamp examination and measurement of intra-ocular pressures.

Inner retinal layer structure was measured using high-resolution spectral-domain optical coherence tomography (SD-OCT; Heidelberg HRA/Spectralis v.6.8.1.0) (Nassif *et al.*, 2004) after pharmacological mydriasis according to a standard protocol (Archibald *et al.*, 2011), as described previously (Leyland *et al.*, 2020). Automatic layer segmentation was applied to compute the thickness of each retinal layer and manually corrected and verified as previously described (Leyland *et al.*, 2020). We focused on the ganglion cell layer (GCL) and inner plexiform layer (IPL) as these are the locations of dopaminergic amacrine cells, with most evidence for thinning in Parkinson’s disease (Chorostecki *et al.*, 2015; Polo *et al.*, 2016) and we have previously shown an association between cognitive risk and these layers in Parkinson’s disease (Leyland *et al.*, 2020).

### Genetic analysis

The dementia risk genes we considered were *APOE4*, *MAPT* (H1/H1), and *GBA*. In our discovery cohort, blood samples were collected, and DNA extracted from an EDTA sample. We performed single nucleotide polymorphism (SNP) array genotyping using the NeuroChip array (Blauwendraat *et al.*, 2017). *MAPT* haplotypes were distinguished using the rs8070723 and rs17649553 SNPs. Standard quality control procedures were conducted to remove individuals with low overall genotyping rates (<98%), related individuals, heterozygosity outliers [>2 standard deviations (SD) from the mean], and population outliers (>6 SD from the mean of any of the first 10 genetic principal components after merging with European samples from the HapMap reference panel). Variants were removed if they had a low genotyping rate (<99%), Hardy-Weinberg equilibrium *P-*value < 1 × 10^–5^_**  **_ and minor allele frequency <1%. Following quality control, genotypes were imputed on the Michigan Imputation Server (https://imputationserver.sph.umich.edu) (Das *et al.*, 2016) to the Haplotype Reference Consortium panel (r1.1).

For the external cohort we downloaded genetics spreadsheets from the PPMI database. SNP genotyping of *APOE* from DNA was performed using the TaqMan™ method, with results in ‘Current_Biospecimen_Analysis_Results.csv’ in the PPMI database. SNP genotyping for *MAPT* and *GBA* was performed using Illumina Immunchip and NeuroX arrays and analysed using Genome Studio v1.9.4 and results in ‘PPMI_PD_Variants_Genetic_Status_WGS_20180921.csv’ in the PPMI database. As only seven patients carried *GBA* mutations in the discovery cohort (*n*** **=** **2 T369M, *n*** **=** **3 E326K and *n*** **=** **2 N370S), these were excluded from the current analyses as *GBA* carriers may have progression that differs in rate and/or sequence (and are likely to have a lower age at dementia onset) but cannot be modelled with a sample of *n*** **=** **7. For consistency we also excluded *GBA* carriers in our PPMI analysis (*n*** **=** **10 controls, *n*** **=** **39 PDD-LR, *n*** **=** **7 PDD-HR). After excluding *GBA* carriers, included patients were tested for genetic variation due to *MAPT* and *APOE4* status in key features of Parkinson’s disease progression (UPDRS-3 and MoCA) using a Mann-Whitney U-test. The Supplementary material contains a detailed analysis of genetics in both cohorts.

### MRI acquisition and image analysis

In the discovery cohort, high-resolution anatomical T_1_-weighted images [magnetization prepared rapid aquisition gradient echo (MP-RAGE)] were acquired at 3 T on a Siemens Prism-fit MRI system with a 64-channel head coil (repetition time = 2530 ms, echo time = 3.34 ms, inversion time = 1100 ms, flip angle α =  7**°**, slices = 176, 1 × 1 × 1 mm voxels, field of view = 256× 256 mm). Susceptibility-weighted MRI images were obtained from a 2 × 1-accelerated, 3D flow-compensated spoiled-gradient-recalled echo sequence. Flip angle 12°; echo time, 18 ms; repetition time, 25 ms; and receiver bandwidth, 110 Hz/pixel. Matrix size was 204 × 224 × 160 with 1 × 1 × 1 mm^3^ voxel resolution (scan time 5 min 41 s). Multishell diffusion weighted imaging (DWI) was acquired with the following parameters: b = 50 s/mm^2^ (17 directions), b = 300 s/mm^2^ (eight directions), b = 1000 s/mm^2^ (64 directions), b = 2000 s/mm^2^ (64 directions); 2 × 2 × 2 mm isotropic voxels, echo time = 3260 ms, repetition time = 58 ms, 72 slices, 2 mm thickness, acceleration factor = 2.

Because of MRI safety requirements, five patients and one control subject were unable to undergo MRI scanning. All images were assessed visually for quality, including artefacts such as motion and distortions. All T_1_-weighted scans were bias-corrected via the FreeSurfer protocol (described in detail in Collins *et al.*, 1994; Dale *et al.*, 1999; Fischl *et al.*, 1999) with parameters optimized for 3 T. Cortical reconstruction and volumetric segmentation of MRI scans for both cohorts was performed using FreeSurfer-v6.0 software (http://surfer.nmr.mgh.harvard.edu/) (Fischl *et al.*, 1999; Fischl and Dale, 2000). This involves skull stripping, volumetric labelling, intensity normalization, grey/white matter segmentation and registration to established surface atlases. Cortical thickness was calculated as the closest distance from the grey–white matter boundary to the grey matter–CSF boundary at each vertex.

Cortical reconstructions were visually inspected slice-by-slice for segmentation errors. Subjects with any inaccuracies that were considered severe enough to significantly affect cortical thickness measures were excluded from the analyses: one control participant failed the reconstruction process and one control’s whole brain was removed from the analysis due to poor segmentation. A further five patients with Parkinson’s disease and three control subjects had the temporal lobe cortical thickness measures excluded due to poor segmentation locally (treated as missing data), but all other brain regions were included for these individuals.

Image acquisition in the PPMI cohort followed a very similar 3D T_1_-weighted 1.5 or 3 T MRI protocol to the local 3 T protocol described above: sagittal plane MP-RAGE or SPGR (spoiled gradient) sequence (slices = 170–200, 1 × 1 × 1.2 mm voxels, 0 mm slice gap, field of view = 256 × 256 mm). Diffusion-weighted images were acquired along 64 uniformly distributed directions with a b-value = 1000 s/mm^2^ and a single b = 0 image (116 × 116 matrix, 2 mm isotropic resolution, repetition time = 900 ms, echo time = 88 ms, 2-fold acceleration). All other parameters followed site-dependent manufacturer recommendations for the scanner used (GE/Siemens/Philips). Scans were read by a radiologist at each site to meet standards of clinical practice and ensure that there were no significant abnormalities. Cortical thickness estimates were obtained as above using FreeSurfer v6.0.0.

#### Quantitative susceptibility mapping reconstruction

QSM image reconstruction was performed as described in Thomas *et al.* (2020). Phase preprocessing used the QSMbox pipeline for single-echo, coil-combined data (https://gitlab.com/acostaj/QSMbox) (Acosta-Cabronero *et al.*, 2018), with 3D complex phase data unwrapped using a discrete Laplacian method. Brain masks (required to separate local from background fields), were calculated from magnitude data using the BET2 algorithm in FSL v5.0. (https://fsl.fmrib.ox.ac.uk). Phase preprocessing was performed using Laplacian boundary value extraction followed by variable spherical mean-value filtering. We estimated susceptibility maps using multi-scale dipole inversion (Acosta-Cabronero *et al.*, 2018). Filtering during reconstruction was performed using an 8 mm kernel. QSM spatial normalization and regional extraction was performed using QSMexplorer (https://gitlab.com/acostaj/QSMexplorer) (Acosta-Cabronero *et al.*, 2016). For template creation, radio-frequency bias corrected MP-RAGE images were spatially normalized using a previously optimized ANTs (http://stnava.github.io/ANTs) routine. Bias-corrected magnitude gradient echo images were then affinely co-registered to their corresponding MP-RAGE volume using ANTs. QSM spatial standardization was completed through a composite warp of the above transformations and high order interpolation. Mean absolute QSM values were extracted from the set of cortical regions defined by the Desikan-Killiany-Tourville atlas (Klein and Tourville, 2012) in OASIS-30 space. The study-wise to OASIS-30 space non-linear warp ﬁeld was calculated with a deformable b-spline co-registration routine in ANTs. Desikan-Killiany-Tourville labels were brought into study space using the inverse of this transformation and nearest-neighbour interpolation. To minimize partial-volume contamination, each cortical region of interest was intersected with a binarized study-wise grey matter mask, inferred from anatomical MP-RAGE images using SPM12 (https://www.fil.ion.ucl.ac.uk/spm/software/spm12/). The PPMI study does not include QSM data.

#### Diffusion weighted imaging reconstruction

Prior to diffusion processing, each volume of the raw data was visually inspected and evaluated for the presence of artefact; only scans with <15 volumes containing artefacts were included (Roalf *et al*., 2016). DWI images underwent standard preprocessing including denoising (Veraart *et al*., 2016), removal of Gibbs ringing artefacts (Kellner *et al*., 2016), eddy-current and motion correction (Andersson *et al*., 2003) and bias field correction (Tustison *et al*., 2010) followed by upsampling to a voxel size of 1.3 mm^3^ (Raffelt *et al*., 2012) and intensity normalization across subjects. Fibre-orientation distributions (FODs) for each participant were computed via multi-shell three-tissue constrained spherical-deconvolution using the group-average response function for each tissue type (grey matter, white matter and CSF) (Dhollander* et al*., 2016). A group-averaged FOD template was created from 30 randomly selected subjects at baseline (20 patients with Parkinson’s disease, 10 healthy control subjects) and each participant’s FOD image was registered to the template (Raffelt *et al*., 2011). Fixel-based metrics were then derived from each subject in template space (Raffelt *et al*., 2012). In addition to fixel-based metrics, we derived the diffusion tensor from each participant’s FOD image (Veraart *et al*., 2013) and calculated a fractional anisotropy (FA) and mean diffusivity (MD) map for each participant, which was registered to template space. Mean fibre cross-section (FC) as well as mean FA and MD were calculated from the left and right substantia nigra based on the DISTAL atlas (Ewert *et al*., 2018). FC was chosen from the three derived fixel-based metrics as prior work by our group, and others, has shown this to be the most sensitive fixel-based metric in both baseline and longitudinal change in Parkinson’s disease (Rau *et al*., 2019; Zarkali *et al*., 2020). Diffusion data from 14 participants in the discovery cohort failed the predetermined quality control criteria and were not included in our analyses (treated as missing data).

For the PPMI cohort we downloaded available diffusion tensor imaging (DTI) metrics from six hand-drawn regions of interest within the left and right substantia nigra (Vaillancourt *et al*., 2009) and included mean FA values.

### Data selection and preparation

We aimed to build a data-driven model of Parkinson’s disease progression in patients at elevated risk for dementia. Most studies of Parkinson’s disease—including the cohorts analysed here—recruit recently diagnosed patients, with very few having severe cognitive deficits. Therefore, as described above, we focused our models on those patients considered to be at elevated risk for developing dementia due to older age at onset (Hietanen and Teräväinen, 1988; Dubois *et al.*, 1990; Jankovic *et al.*, 1990; Biggins *et al.*, 1992; Katzen *et al.*, 1998; Aarsland *et al.*, 2001, 2007; Akinyemi *et al.*, 2008; Williams-Gray *et al.*, 2013). Specifically, we use a threshold age at onset of 65 years, classifying older patients at diagnosis (≥65 years old) as PDD-HR, and the other patients as PDD-LR. Our threshold reflects the average age at onset for Parkinson’s disease, which is estimated to be between 60 and 70 years old (Macleod *et al.*, 2018). We stress-tested this dementia risk threshold by building models using thresholds of 60 and 70 years, which showed strong and significant rank correlation with the main model presented (Supplementary material). Table 1 shows detailed demographics of included participants in our cohorts.

In our discovery cohort we selected clinical, cognitive, retinal, and visual measures from our battery (Leyland *et al.*, 2020) based on specificity to Parkinson’s dementia (Liu *et al.*, 2017; Schrag *et al.*, 2017; Leyland *et al.*, 2020). UPDRS scores were not included in the modelling because motor symptoms are present in all patients with Parkinson’s disease (by definition). Measures of average cortical thickness in each brain lobe (five per hemisphere: frontal, parietal, occipital, temporal, plus the cingulate) were calculated from T_1_-weighted structural MRI by combining the default estimates returned by FreeSurfer for the 68 cortical regions of interest in the Desikan-Killiany atlas (Desikan *et al.*, 2006). We included absolute QSM measures from 12 brain regions out of 96 in the Desikan-Killiany-Tourville atlas using the Mann-Whitney U-test for PDD-HR versus controls (*P*** **<** **0.05 uncorrected). We adjusted all measures for gender, age, and years of education using a linear model: *y* ∼ *gender* × (*age + education*) trained on controls. This accounts for confounding effects such as the influence of age and education on performance in clinical assessment tasks and allows for possible differences between genders.

For our comparison experiments on PPMI data we included a subset of measures: only those available in both cohorts (see text and Supplementary material).

### Statistical analysis

#### The event-based model

**Figure 1:**
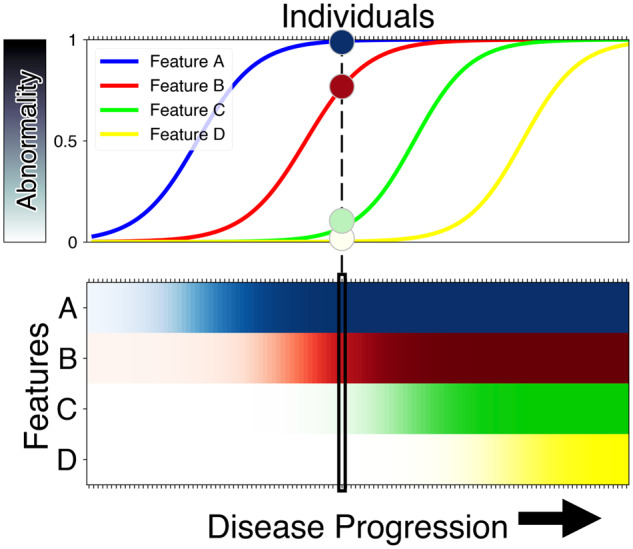
**How the event-based model works.** The event-based model is a statistical method for quantifying a sequence of observable abnormality in a set of disease-relevant features (biomarkers). The model works by assessing, at the group level, combinations of simultaneously normal and abnormal measurements in different biomarkers across individuals at multiple stages of disease progression. *Top*: In neurodegenerative disease progression (left to right), observable abnormality (vertical axis) across multiple features (A, B, C, D) likely proceeds in a cascade or sequence A→ B→C→D, as in an influential hypothetical model of Alzheimer’s disease progression (Jack *et al.*, 2010; Jack Jr *et al.*, 2013). *Bottom*: A cross-sectional sample of individuals (columns) at different stages of disease progression (horizontal axis) showing the corresponding observed combinations of normal (white) and degrees of abnormality (shades of colour) across the four features. A single individual sampled near the middle of the disease is shown in both panels: early events (A and B) have higher abnormality than later events (C and D). Whenever such an individual shows an elevated value of biomarker A, but a normal value for biomarker B, this adds evidence that A changes before B.

We updated a recent version of the event-based model that incorporates non-parametric mixture modelling (Firth *et al.*, 2020). Our novelty is described below. We used this model to estimate the most likely sequence of clinical and neuroimaging events in the progression to Parkinson’s disease dementia, and the uncertainty (positional variance) in the sequence. This longitudinal picture of disease progression is extractable from cross-sectional data by assuming a single monotonic progression and exploiting combinations of observable abnormality (earlier/later events) across individuals (Fig. 1). By analogy, if all patients who present with the common cold have a cough, but only some of these also sneeze, we would infer with very high confidence that coughing comes before sneezing (of course, this analogy only works for a hypothetical common cold that is progressive/non-remitting). The confidence in the ordering of events is quantified intrinsically within the event-based model and depends on the data (and the nature of disease progression). Returning to our analogy, confidence in the ‘cough-then-sneeze’ sequence would be reduced if only most patients presented in this manner, rather than all patients. We present event-based models as positional density heat maps, also known as positional variance diagrams (Fonteijn *et al.*, 2012). A positional density heat map shows the posterior distribution of events and their position in the most likely sequence. ‘Hot’ regions of high density correspond to high confidence in the ordering, appearing as a narrow, dark diagonal pattern in the map. ‘Cool’ regions of broad, lower density correspond to lower confidence in the ordering, e.g. some events are likely to occur concurrently as far as the model can discern from the data. Our methodological novelty is in the mixture modelling step of event-based modelling. We allowed only a small fraction of data from controls to be labelled as abnormal/post-event. Specifically, any controls having extreme abnormality above the 90th percentile for that marker (including control and patient data) were labelled as abnormal, otherwise data from controls remained labelled as normal/pre-event. This adds clear interpretability to our models: they represent progression to Parkinson’s dementia as disease-specific deviations from normality. Markers were excluded if mixture modelling was unable to discern disease signal from the data (separation between controls and patients consistent with disease progression).

#### Similarity measure for event-based models

We use the Bhattacharrya coefficient (*BC*) as a similarity measure (Liese and Miescke, 2008) for event-based models in terms of statistical overlap between two posterior distributions:

(1) $$BC=1-H^{2}$$

where *H* is the mean Hellinger distance (Watanabe, 2009) between models, calculated row-wise in the positional density map (mean and SD reported). The Bhattacharrya coefficient is equal to zero when the posterior positional densities of two event-based models do not overlap (maximal Hellinger distance), and it is equal to unity when the positional densities overlap exactly. To provide some context, we calculate a reference value of *BC_0 _*= 0.37 ± 0.02, which represents the statistical similarity of randomized models (Supplementary material).

#### Patient staging

Each individual participant is assigned a fine-grained disease stage within the model via the data likelihood for their set of measurements across events. Following Young *et al.* (2014), we assigned the stage that maximizes the individual likelihood. Patient stage is akin to a data-driven, multivariate risk score, with previous work showing strong predictive value for cognitive outcomes in sporadic Alzheimer’s disease (Young *et al.*, 2014), and for disease onset in familial Alzheimer’s disease (Oxtoby *et al.*, 2018) and Huntington’s disease (Wijeratne *et al.*, 2018).

#### Cross-validation

We used repeated stratified 5-fold cross-validation to ensure robustness of the event-based model results. This involved refitting both the mixture models and the sequence on 80% of the cohort data and testing accuracy on the held out 20% for each of 10 5-fold random partitions, giving a total of 50 cross-validation folds/models, which are averaged to find the final model (see Supplementary material for details). This gives a more robust model (both in terms of the sequence and uncertainty in the sequence) than a single maximum-likelihood model built on 100% of the data. Additionally, this enables us to compare the 50 models to assess model robustness and generalizability using cross-validation similarity and consistency quantified by the Bhattacharyya coefficient given above. We cross-validated model accuracy via patient staging (described above) with the gold standard model stage provided by staging from the full cross-validated model (all folds combined). This necessitated two runs of cross-validation: one to generate the ground truth model stage for each participant, and a second to calculate metrics across cross-validation folds. Model robustness is evidenced by high similarity (average *BC* closer to unity) and simultaneously high consistency (low standard deviation of *BC*) between models across cross-validation folds. Model accuracy is evidenced by low errors in patient staging across folds.

#### External cohort: PPMI

For comparison, our experiments were repeated on available data in the PPMI cohort. We included healthy controls and patients with Parkinson’s disease (Table 1). Our PPMI experiments included the subset of measures available: clinical, cognitive, DTI metrics in the substantia nigra, and cortical thickness. Our retinal and visual measures are not available in PPMI, nor are the brain iron content biomarkers from QSM. We quantify the comparison statistically using rank correlation and our similarity measure defined above after first averaging the posterior within data modality (Supplementary material).

### Data availability

The derived data and python code that support the findings of this study are available from the corresponding author, upon reasonable request. The underlying event-based model code is publicly available at https://github.com/noxtoby/kde_ebm_open.

## Results

### Participants

Table 1 presents key descriptors in both cohorts. Briefly, our discovery cohort included 33 controls and 36 PDD-HR patients. These groups were used to estimate event distributions within the event-based model, i.e. marker/event severity in the progression to Parkinson’s dementia. See Supplementary material for details of the event distributions. All patients (including 64 PDD-LR) were used to estimate the sequence of events (under the assumption that all patients would eventually progress to dementia, given sufficient survival). Our external cohort included 127 controls, 146 PDD-HR patients, and 206 PDD-LR patients. In MoCA and UPDRS-3 scores we found no variation due to *MAPT* or *APOE4* status (Mann-Whitney U-test, all *P* ≥ 0.13) and, when *GBA* cases were included in the analyses, results were statistically indistinguishable (Supplementary material). Together this suggests that genetic variation is not a factor in our study (larger numbers are required to investigate progression in genetic Parkinson’s, particularly for *GBA*).

### Features: clinical, cognitive, visual and imaging markers

**Figure 2:**
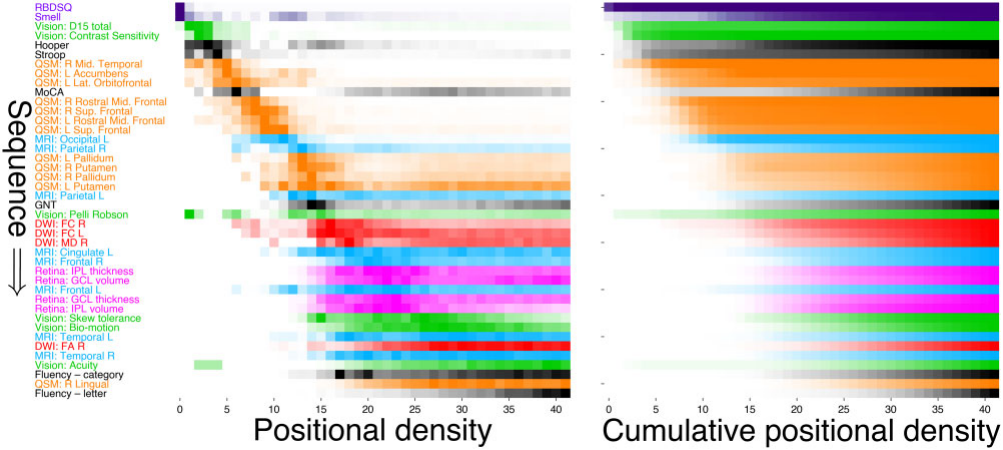
**Event-based model of progression in Parkinson’s disease.** Data-driven sequence of events in Parkinson’s disease progression colour coded by modality shown as: positional density (*left*); and cumulative abnormality (*right*) from repeated stratified 5-fold cross-validation. The estimated sequence of events is seen on the vertical axis, with ordering proceeding from top to bottom (earliest to latest event). Colour intensity represents the proportion (0 in white, 1 most intense) of the posterior distribution in which events (*y*-axis) appear in a particular position in the sequence (*x*-axis). This model is robust, having a similarity of *BC* = 0.60 ± 0.04 across 50 cross-validation folds. D15 = colour test; GCL = ganglion cell layer; GNT = Graded Naming Test; IPL = inner plexiform layer; L = left; R = right; RBDSQ = REM sleep behaviour disorder screening questionnaire; UPDRS = Unified Parkinson’s disease rating scale.

Our final set of 42 features for discovery is shown on the vertical axis of Fig. 2, including eight clinical/cognitive measures, six vision measures, four retinal measures, eight regional measures of cortical thickness, four measures of white matter neurodegeneration in the substantia nigra, and 12 regional measures of brain iron content. The subsets of features for cross-cohort comparison are shown on the vertical axes of Fig. 4, including clinical/cognitive measures, measures of white matter neurodegeneration in the substantia nigra, and measures of regional cortical thickness.

### Sequence of events in Parkinson’s disease progression

Our cross-validated probabilistic model of Parkinson’s disease progression in patients at elevated risk of dementia is presented as the positional density map in Fig. 2 (left). The right side shows a complementary visualization of the cumulative abnormality (left to right). In each panel, disease progresses from upper left to lower right, with colour intensity reflecting row-wise positional/cumulative density and confidence in the ordering. Thus, a dark diagonal pattern (left panel) shows strong confidence in the ordering and a light, off-diagonal pattern shows weak confidence in the ordering. Rows in Fig. 2 are coloured to reflect the data modality.

The model estimates that progression starts with classic prodromal features of Parkinson’s disease (REM-sleep behaviour problems, olfactory dysfunction; shown in purple) then proceeds to early visual dysfunction (D15 colour, contrast sensitivity; light green), then increased iron content primarily in temporal and frontal regions (orange) along with cognitive deficits (Stroop, Hooper, MoCA; greyscale), then abnormal cortical thickness apparently starting in the occipital lobe (light blue). At this point the ordering becomes less certain, but involves white matter neurodegeneration in the substantia nigra (red), retinal thinning (magenta), further cortical neurodegeneration, iron accumulation, vision deficiency (in tests of skew tolerance, biological motion, and acuity), and cognitive decline (fluency and language dysfunction).

### Patient staging

**Figure 3:**
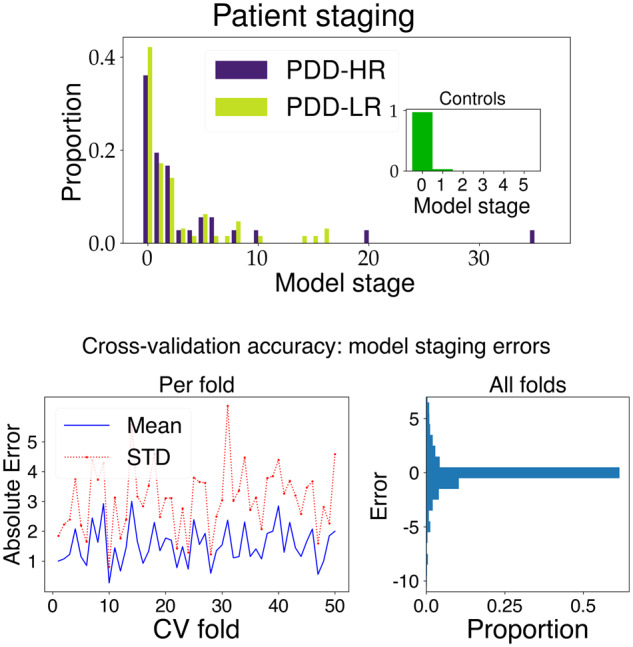
**Patient staging results: discovery cohort.** *Top*: Model stage showing most healthy controls at stage zero (*inset*); and patients at varying, but mostly early, stages. *Bottom*: Cross-validation accuracy across 50-folds from repeated stratified 5-fold cross-validation. *Left*: Mean and standard deviation (STD) absolute error in patient stage. *Right*: raw errors in patient stage. Overall mean absolute error was 1.5 ± 3.3 stages. CV = cross-validation.

We assigned each participant in the discovery cohort to their most likely numerical stage within the model (see ‘Materials and methods’ section and Young *et al.*, 2014), given their data. A histogram of the staging results is shown in Fig. 3. Qualitatively, model stage concurs with expectations, i.e. healthy controls (Fig. 3) are very early (mostly stage zero) with Parkinson’s disease patients at varying stages (we found no statistically significant differences between PDD-LR and PDD-HR stages).

### Cross-validation

Figure 3 shows that, under cross-validation, the model is likely to generalize well to other patient cohorts: good similarity (high mean *BC*), high consistency (low SD *BC*), and high accuracy (low staging errors) across 50-folds. Ten repeats of stratified 5-fold cross-validation produced high statistical overlap of *BC* = 0.60 ± 0.04 (mean, SD across folds) relative to the reference value *BC_0_* = 0.37 ± 0.02 (Supplementary material), as well as low mean absolute error of 1.5 ±_** **_3.3 stages in patient staging (see ‘Materials and methods’ section).

### Comparison with external dataset: PPMI

**Figure 4:**
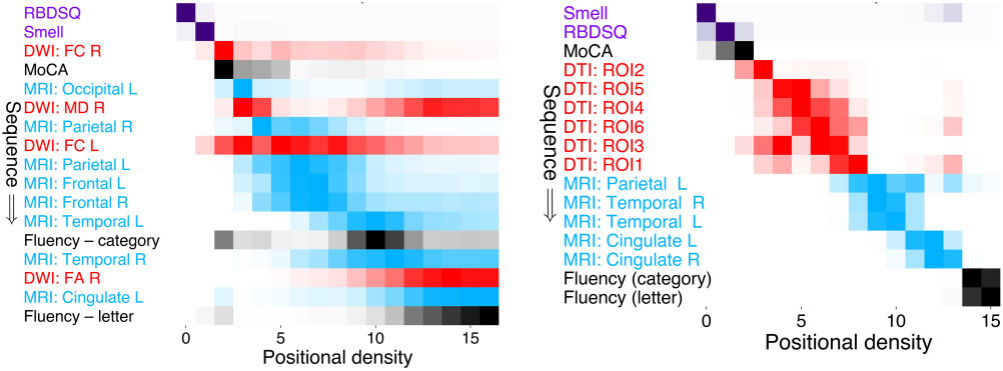
**Visual comparison of progression models in the discovery and external cohorts.** Event-based models of Parkinson’s disease progression built on a subset of comparable clinical/cognitive/MRI features in the local cohort (*left*) and PPMI cohort (*right*). Both models suggest that Parkinson’s disease starts with classic symptoms of prodromal Parkinson’s disease (sleep/smell abnormality), with neurodegeneration occurring later in the substantia nigra (DWI/DTI) and cortex (MRI). Cognitive abnormality appears first in the MoCA score, preceding measures of verbal fluency.

A visual comparison of our Parkinson’s progression model (Fig. 2) with a model built using the PPMI dataset is shown in Fig. 4, on the subset of comparable features available in both cohorts. The models show high qualitative and quantitative concordance: classical Parkinson’s symptoms and cognitive decline precedes neurodegeneration, with white matter degeneration in the substantia nigra generally preceding cortical thinning; supported by high rank correlation τ =  0.87 (*P*** **=** **0.017) and statistical similarity *BC* = 0.96 (Supplementary material). Supplementary Fig. 5 explores the subsequence of abnormality in cortical thickness.

## Discussion

We used data-driven event-based modelling to reveal the fine-grained probabilistic sequence of decline in clinical, cognitive and neuroimaging measures during Parkinson’s disease. We enriched for Parkinson’s dementia by focusing the model on patients at high risk of dementia due to their older age at onset (≥65 years) and used repeated stratified cross-validation for robustness, plus repeat experiments on an external cohort for comparison. Our dementia enrichment strategy is supported by the PDD-HR group having significantly lower MoCA scores than the PDD-LR group at follow-up ∼1.5 years later (Mann-Whitney U-test, *P*** **<** **0.05; Supplementary material).

Our data-driven model estimates that the earliest events in Parkinson’s disease progression in patients at elevated risk of dementia include measures of REM sleep behaviour problems and olfactory dysfunction. These events are followed by early changes in visual performance (including colour vision loss), and cognitive dysfunction. The earliest events detectable with neuroimaging are QSM estimates of brain tissue iron accumulation in frontal and temporal regions, before regional abnormality in cortical thickness from T_1_-weighted MRI and white matter neurodegeneration in the substantia nigra from diffusion-weighted MRI. The data and model suggest that retinal thinning, like cortical thinning, is a relatively late occurrence. Our results provide data-driven support for current understanding of Parkinson’s disease progression, with early loss of smell and REM sleep changes (Hummel *et al.*, 2007; Stiasny-Kolster *et al.*, 2007). As our aim was to model progression in established disease, all patients had Parkinson’s disease (rather than prodromal disease), which is why we omitted UPDRS scores from the model. Including them would see motor dysfunction as the earliest event, which could be somewhat misleading at the individual patient level since studies of populations in prodromal stages of Parkinson’s disease have shown that smell and sleep changes are often found before motor changes (Korczyn and Gurevich, 2010). Large longitudinal cohorts that include the prodromal phase in confirmed cases will be useful to confirm the ordering of these early premotor features.

We highlight the relatively early appearance of colour detection deficits and contrast sensitivity, measured here by simple tests capable of being performed by opticians and optometrists. Early involvement of visual dysfunction is interesting—particularly so because it precedes retinal abnormality and the earliest cortical neurodegeneration. This is consistent with emerging data that suggest visual changes are an early manifestation or even a precursor of cognitive change in Parkinson’s disease (Williams-Gray *et al.*, 2013; Anang *et al.*, 2014), including some of our own work on the discovery cohort (Leyland *et al.*, 2020) and more recently showing, in very large cohorts, that Parkinson’s disease patients with poor vision have a worse phenotype with higher rates of dementia (Hamedani *et al.*, 2020; Han *et al.*, 2020). Together, this evidence suggests the exciting prospect that routine assessment of vision may have a role in disease stratification for cognitive decline in Parkinson’s disease.

Our model placed abnormal measures of brain tissue iron before abnormal cortical thickness in Parkinson’s disease progression. We also found that the sequence of regional cortical thinning was highly uncertain (Supplementary material). This is consistent with reports that grey matter atrophy is inconsistent across patients and often a late event in Parkinson’s dementia (Hattori *et al.*, 2012; Lanskey *et al.*, 2018) and is also consistent with our recent separate work on the discovery cohort showing that cortical brain iron increases are seen in Parkinson’s disease patients in relation to cognitive performance (Thomas *et al.*, 2020). The heterogeneity observed in both cohorts motivates future work on data-driven subtyping (see below).

Our model suggests that loss of retinal tissue in key dopamine-containing layers (GCL and IPL) occurs after brain tissue iron increases and grey and white matter atrophy has commenced. This might suggest that retinal changes are likely to occur later, after retrograde signals from cortical brain regions, and not concurrently with brain atrophy. Similar effects of cortical damage having a retrograde effect on retinal thickness are seen in multiple sclerosis (Henderson *et al.*, 2008). This could be verified in prospective datasets. Recent models of axonal degeneration as an early feature of Parkinson’s disease suggest that this process of de-arborization may occur throughout the nervous system, and could explain changes seen in both the retina and cortex (Adalbert and Coleman, 2013), but with differing timings.

A particular strength of our study is the analysis of an external dataset which supports many of our conclusions on the subset of features available in both cohorts. Specifically, models built on each cohort showed quantitative agreement on the ordering of prodromal symptoms of Parkinson’s disease, cognitive decline, white matter neurodegeneration in the substantia nigra, and cortical thinning (Supplementary material). This is remarkable given the considerable difference in age and disease duration between the cohorts: PPMI patients are younger and have less advanced disease (Table 1), due to our cohort being enriched for older onset Parkinson’s disease and also for longer disease duration.

We did not find differences in progression features between patients carrying *APOE4* or *MAPT* H1/H1 polymorphisms in our cohort or the PPMI dataset and our analyses were not powered to detect differences in the sequence of progression between groups carrying these polymorphisms. We also excluded the small number of patients carrying *GBA* mutations as these patients are likely to have a more rapid progression to dementia and may show a divergent sequence of events (Blauwendraat *et al.*, 2020), although we note that the model is statistically unchanged if these seven patients are included [τ =  0.75 (*P*** **=** **2 × 10^–12^_** **_) and *BC* = 0.99] (Supplementary material). Future work including much larger numbers of patients, and enriched for specific genetic subtypes, should specifically examine the role of genetic variation in modifying the rate and sequence of events in Parkinson’s disease.

The event-based model would also be of interest applied to dementia with Lewy bodies or established Parkinson’s disease dementia. As visual processing deficits are seen in the prodromal phase of dementia with Lewy bodies (McKeith *et al.*, 2020) and in patients with more rapid Parkinson’s disease dementia (Anang *et al.*, 2014; Hamedani *et al.*, 2020), we predict that visual changes will also be found as early events in Lewy body dementia.

### Limitations and future work

In the absence of markers of disease progression for prodromal Parkinson’s dementia, we used later age at onset (≥65 years) as a proxy for a higher-risk group for Parkinson’s dementia. This is based on a wealth of evidence that higher age at onset of Parkinson’s disease is a strong predictor for earlier and more aggressive Parkinson’s dementia (Dubois *et al.*, 1990; Katzen *et al.*, 1998) and is likely to be a more robust marker than, for example, global cognitive scores such as the MMSE or MoCA, which lack sensitivity in diagnosing dementia in Parkinson’s disease (Zadikoff *et al.*, 2008; Hoops *et al.*, 2009), particularly at early stages of Parkinson’s dementia. Ultimately, quantitative markers of disease activity in Parkinson’s dementia are needed to enable early detection and better stratification of patients at risk of Parkinson’s dementia. Our data-driven model may fill this role, being akin to a multimodal, computational biomarker of progression to Parkinson’s dementia.

Our results are built on cross-sectional data from patients with established Parkinson’s disease, at elevated risk of developing Parkinson’s dementia. In order to fully validate our results, we aim to test the model on prospective data, with enough follow-up time to allow for conversion of patients to Parkinson’s dementia. This is ongoing work. Indeed, our models could be used to inform the design of such prospective studies, e.g. identifying which events are involved early/late in the progression to Parkinson’s dementia.

The event-based model assumes a single sequence in the progression, which is unrealistic given the widely documented variability in presentation of Parkinson’s disease patients. This limitation means that we cannot necessarily distinguish between events that occur early in only a proportion of patients and events that occur late in all patients, both of which would appear with relatively low frequency in a patient population. Indeed, our experiments provided evidence for such heterogeneity—manifested as off-diagonal positional density in Figs 2 and 4. Addressing this limitation is the topic of future work.

We have plans for multiple lines of future work motivated to further improve understanding of Parkinson’s disease progression. First, we are actively seeking access to data from suitable cohorts in order to characterize the prodromal phase. Second, we aim to unravel the aforementioned heterogeneity through automatic data-driven subtyping of disease progression which has had success in other dementias (Young *et al.*, 2018) and chronic obstructive pulmonary disorder (Young *et al.*, 2020). Finally, we are keen to understand biological mechanisms in Parkinson’s disease and other neurodegenerative diseases, for which topological profile models that examine the patterns of atrophy and network involvement (Garbarino *et al.*, 2019) are a promising method.

### Summary

We used data-driven event-based modelling to determine that the most likely sequence of events in Parkinson’s disease progression in patients at elevated risk of dementia begins with classic prodromal features of Parkinson’s disease followed by early visual deficits and some cognitive dysfunction, increased brain iron content, then neurodegeneration in the substantia nigra, cortex, and retina, and further decline.

The models we have constructed, and those we aim to construct in the future, should prove useful in clinical practice and informing the design of future studies. For example, the fine-grained patient staging mechanism utilized in this study has the potential to reduce heterogeneity in a patient cohort and to support precision medicine decisions. Likewise, we expect that our future data-driven subtyping work will be able to reduce heterogeneity further, as demonstrated in other dementias (Young *et al.*, 2018). Ultimately, we are convinced that these computational models, as part of a larger effort, offer a tangible route towards identifying a disease-modifying therapy in Parkinson’s disease.

## Supplementary Material

awaa461_Supplementary_Data

## Abbreviations

PDD-HR =: Parkinson’s disease dementia-high risk
PDD-LR =: Parkinson’s disease dementia-low risk
MoCA =: Montreal Cognitive Assessment
QSM =: quantitative susceptibility mapping
PPMI =: Parkinson’s Progression Markers Initiative
