# Supplementary material for: Sequence of clinical and neurodegeneration events in Parkinson’s disease progression
Source: Brain. 2021 Feb 5;144(3):975–88. doi: 10.1093/brain/awaa461 (PMC8041043; doi:10.1093/brain/awaa461)
Supplement: awaa461_Supplementary_Data [file awaa461_supplementary_data.pdf]

Supplementary Material

Sequence of clinical and neurodegeneration events in Parkinson's disease progression

Neil P. Oxtoby, et al.

In this document we provide additional detailed information on the discovery and external cohorts; mixture modelling for event probabilities in the event-based model; combining cross-validation folds into a final event-based model; present additional results of our validation experiments and a cross-cohort model comparison; analyse cognitive decline in patient groups enriched for dementia risk; examine dementia-specific variation with *ApoE* and *MAPT*; and estimate the position of dysautonomia in Parkinson's disease progression using the external cohort.

Table of Contents

1. COHORT COMPARISON ... 2

2. MIXTURE MODELS FOR EVENT PROBABILITIES ... 3

3. MODEL VALIDATION ... 4

3.1. COMBINING EVENT-BASED MODELS FROM CROSS-VALIDATION ... 4
3.1.1. CROSS-VALIDATION SEQUENCE ... 4
3.1.2. SIMILARITY REFERENCE: RANDOMISED MODELS ... 5
3.2. CROSS-VALIDATION: SUPPLEMENTARY RESULTS ... 6

4. ROBUSTNESS TO PDD-HR THRESHOLD (AGE AT ONSET) ... 6

5. CORTICAL THINNING MODEL ... 8

6. COGNITIVE DECLINE IN PATIENTS ENRICHED FOR DEMENTIA RISK ... 9

7. GENETIC VARIATION ... 9

8. CROSS-COHORT MODEL COMPARISON ... 10

9. DYSAUTONOMIA IN PARKINSON'S PROGRESSION: SCOPA-AUT ... 11

1. Cohort comparison

Supplementary Table 1. **Cross-cohort comparison: discovery (local); external (PPMI). Cross-cohort comparison, within groups.**
Each *p* value shown is for a Mann-Whitney *U*-test (median) or χ^2-test (proportion) of the null hypothesis that there is no statistical difference between the cohorts, within each clinical group: Controls, PDD-HR, PDD-LR. Comparisons of olfaction between the cohorts is made possible by converting UPSIT (PPMI) to a Sniffin' Sticks equivalent score using an equipercentile method (Lawton *et al.*, 2016).
Abbreviations: PD – Parkinson's disease; PDD-HR – PD dementia high-risk; PDD-LR – PD dementia low-risk; UPDRS – Unified PD Rating Scale; F/M – female/male; RBDSQ – REM Sleep Behaviour Disorder Screening Questionnaire; MoCA – Montreal Cognitive Assessment; GNT – Graded Naming Test. MAPT +/- refers to presence versus absence of H1/H1 status. APOE4 +/- refers to presence or absence of at least 1 APOE4 allele.

**Controls**	**Discovery (N=33)**	**External (N=127)**	***U* or χ^2**	***p***
Age, years	64.7 (9.0)	60.0 (11.0)	1613.5	0.021
UPDRS total	8.2 (5.2)	1.8 (2.8)	471.5	< 0.0001
Gender F/M	18/15	48/79	2.38	0.123
RBDSQ	1.7 (1.3)	1.9 (1.4)	1891.5	0.190
Olfaction (Sniffin' Sticks equivalent)	12.2 (2.6)	13.6 (1.7)	1329	0.0005
Cognition (MoCA)	28.6 (1.3)	28.2 (1.1)	1797	0.098
Category Fluency (animals)	22.1 (5.2)	22.0 (5.4)	2094	0.498
MAPT +/–	11/6	51/75	2.66	0.103
APOE4 +/–	3/14	27/90	0.036	0.849
**PDD-HR**	**Discovery (N=36)**	**External (N=146)**	***U* or χ^2**	***p***
Age, years	73.0 (3.9)	70.9 (3.7)	1849	0.003
Disease duration, years	3.4 (2.1)	0.65 (0.62)	453	<0.0001
Age at onset PD	70.1 (3.8)	70.3 (3.7)	2554.5	0.398
UPDRS total	42.9 (18.1)	30.2 (11.1)	1482.5	<0.0001
Gender F/M	13/23	48/98	0.029	0.864
RBDSQ	4.2 (2.4)	4.2 (2.6)	2578.5	0.431
Olfaction (Sniffin' Sticks equivalent)	6.8 (3.4)	7.4(3.6)	2353.5	0.166
Cognition (MoCA)	27.6 (2.2)	26.8 (2.2)	2052	0.020
Category Fluency (animals)	20.2 (6.4)	19.5 (4.6)	2518	0.349
MAPT +/–	14/8	50/92	5.3	0.021
APOE4 +/–	7/15	30/101	0.403	0.526
**PDD-LR**	**Discovery (N=64)**	**External (N=206)**	***U* or χ^2**	***p***
Age, years	59.7 (5.1)	55.6 (7.3)	4545.5	<0.0001
Disease duration, years	4.7 (2.6)	0.53 (0.55)	421.5	<0.0001
Age at onset PD	55.5 (4.4)	55.0 (7.3)	6319	0.309
UPDRS total	46.8 (24.0)	26.0 (11.1)	2623.5	<0.0001
Gender F/M	35/29	72/134	7.15	0.008
RBDSQ	4.2 (2.5)	3.9 (2.5)	6062	0.164
Olfaction (Sniffin' Sticks equivalent)	8.1 (2.9)	9.2 (3.4)	5277	0.008
Cognition (MoCA)	28.2 (1.7)	27.4 (2.2)	5257.5	0.006
Category Fluency (animals)	22.0 (5.1)	21.8 (5.5)	6325.5	0.313
MAPT +/–	30/11	69/132	19.7	<0.0001
APOE4 +/–	6/35	45/134	1.52	0.218

Supplementary Table 1 is a summary comparison of our discovery and external cohorts. We compare the cohorts statistically within groups: controls, PDD-LR and PDD-HR. Highlights include the following:

- The PPMI cohort is younger on average (but we adjusted for age effects).
- PPMI patients had a shorter disease duration (and lower UPDRS scores), but the patient groups in both cohorts had comparable age at onset of symptoms.
- Our discovery cohort controls had more females (but we adjusted for gender effects).
- PPMI controls (and younger patients, PDD-LR) had worse olfaction.
- PPMI patients had slightly lower MoCA scores.

Exclusions. We reported exclusions in the main manuscript for our Discovery cohort. Those excluded for confounding neurological conditions included possible Multiple System Atrophy (n=2); Progressive Supranuclear Palsy (n=1); epilepsy (n=1). See https://ppmi-info.org for information on inclusion/exclusion criteria in the external cohort (PPMI).

2. Mixture models for event probabilities

Patients have different marker profiles that reflect disease severity, e.g., different scores on clinical tests and different severity/extent of neurodegeneration. The event-based model exploits this fact to infer a longitudinal sequence of events from a cross-sectional sample by assessing marker severity across individuals. Severity is quantified using a mixture model that assigns “normal” and abnormal” labels to each individual’s marker data (be they patient or control) separately. This is done in a data-driven fashion, usually starting with all data from controls assigned the “normal” (“pre-event”) label and data from patients assigned the “abnormal” (“post-event”) label, with labels swapped for outliers in each group until convergence. In the original event-based model demonstrated in sporadic Alzheimer’s disease (Young *et al.*, 2014), the mixture modelling was implemented by allowing only patient labels to be swapped, under the assumption that controls are extremely unlikely to be prodromal patients due to stringent inclusion criteria. In this work we take the same “fixed-controls” mixture modelling approach but allow for outliers to swap labels — marker data from controls that is in the 90th percentile or above (patients and controls together) is allowed to participate in the label swapping of the mixture modelling. We also inform the mixture modelling with prior information on disease direction for each biomarker. We encode these innovations within the nonparametric mixture modelling approach introduced in (Firth *et al.*, 2020) and available at https://github.com/noxtoby/kde_ebm_open. Together, our innovations add clear interpretability to our models: they represent Parkinson’s disease progression in patients at elevated risk of dementia as disease-specific deviations from normality.

Supplementary Figure 1 shows histograms and representative mixture models for selected features included in our model (Figure 2, main manuscript). We include at least one of each feature type: classic PD symptoms, visual tests, cognitive tests, QSM, MRI, DWI, retinal measures.

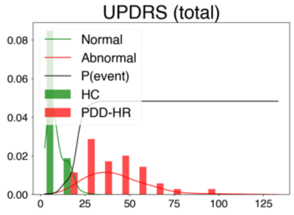

UPDRS total score

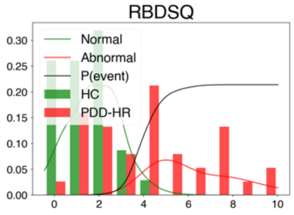

RBDSQ

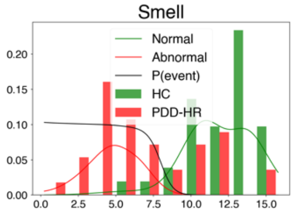

Smell

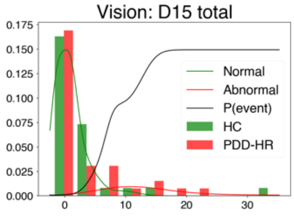

Vision: D15 total errors (colour)

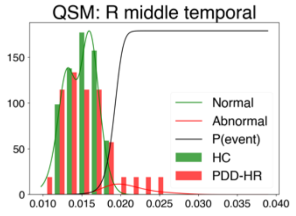

QSM: right middle temporal

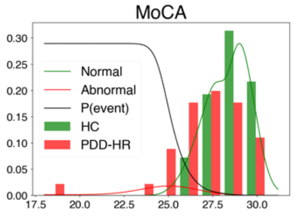

MoCA

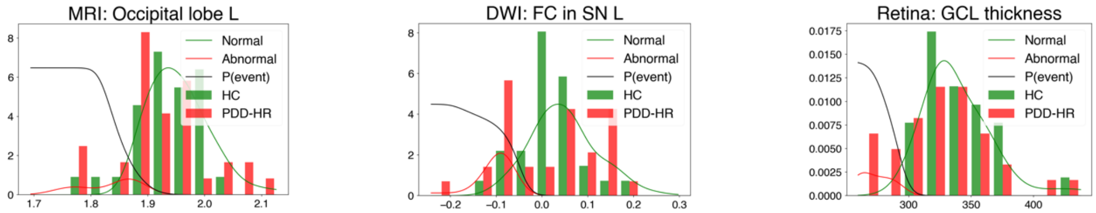

MRI: right occipital lobe | DWI: FC in left substantia nigra | Retina GCL thickness

Supplementary Figure 1. ***Event distributions from our novel mixture-modelling approach.*** *Exemplars of our model's event severity scores using kernel density estimate mixture models (Firth et al., 2020). Data shown has been detrended for gender, age and years of formal education as described in the main manuscript.*

3. Model Validation

3.1. Combining event-based models from cross-validation

An event-based model consists of a sequence and uncertainty in that sequence. This is represented by a square probability matrix, with the jth row being the posterior density for biomarker j over sequence positions k. We call this a positional density map, or a positional variance diagram (Fonteijn *et al.*, 2012). Typically, the sequence is determined using maximum-likelihood estimation, and the density is determined by multiply initialised MCMC samples from the posterior.

In this paper we used 10-repeated 5-fold cross-validation which produces 50 event-based models, each trained on different partitions of 80% of the data. We average the posteriors of these 50 models to form our cross-validated posterior density map.

3.1.1. Cross-validation sequence

Aggregating the 50 model sequences is not straightforward because rank aggregation is not a solved problem and depends on the context, e.g., voting (Arrow, 1950), and event-based modelling (Huang and Alexander, 2012; Venkatraghavan *et al.*, 2019). Rather than averaging the 50 model sequences, we introduce the concept of "event onset" to define the CV (cross validation) sequence. This is motivated by the original hypothetical models of dynamic biomarkers in Alzheimer's disease (Aisen *et al.*, 2010; Frisoni *et al.*, 2010; Jack *et al.*, 2010) which inspired the original event-based model. The hypothesis is that biomarker *change*/*dynamics* occurs in a sequential manner. We quantify biomarker change using the derivative of the cross-validated *cumulative* positional density map (row-by-row for each event separately), then use this to define event onset as the point of maximum rate-of-change, subject to reaching a minimum cumulative abnormality of $C > 0.25$. We also use the value of cumulative abnormality as a tiebreaker, with higher cumulative abnormality ranking an event earlier. If the tie remains, then we rank higher the earliest marker to reach 50% cumulative abnormality using $argmin_s |C_j(s) - 0.5|$, where s is the sequence position.

Mathematically, event onset o_j for marker j is the sequence position that maximises the derivative of posterior cumulative positional density C_j

$$o_j = argmax_s \frac{dC_j}{ds} \quad , \qquad s.t.\ C_j > 0.25$$

We illustrate this in Supplementary Figure 2 using our full model from Figure 2 in the main manuscript. Supplementary Figure 2A is a representation of the right panel of Figure 2: the cumulative positional density map consisting of curves C_j for events j (curves smoothed using kernel density estimation for visualisation purposes). Supplementary Figure 2B shows

the corresponding derivatives dC_j/ds with red dots showing event onset as defined above. Supplementary Figure 2C is a 2D scatter plot of cross-validated sequence position against event onset, which reemphasises the uncertainty found later in the model sequence (see discussion around Figure 2 in the main manuscript).

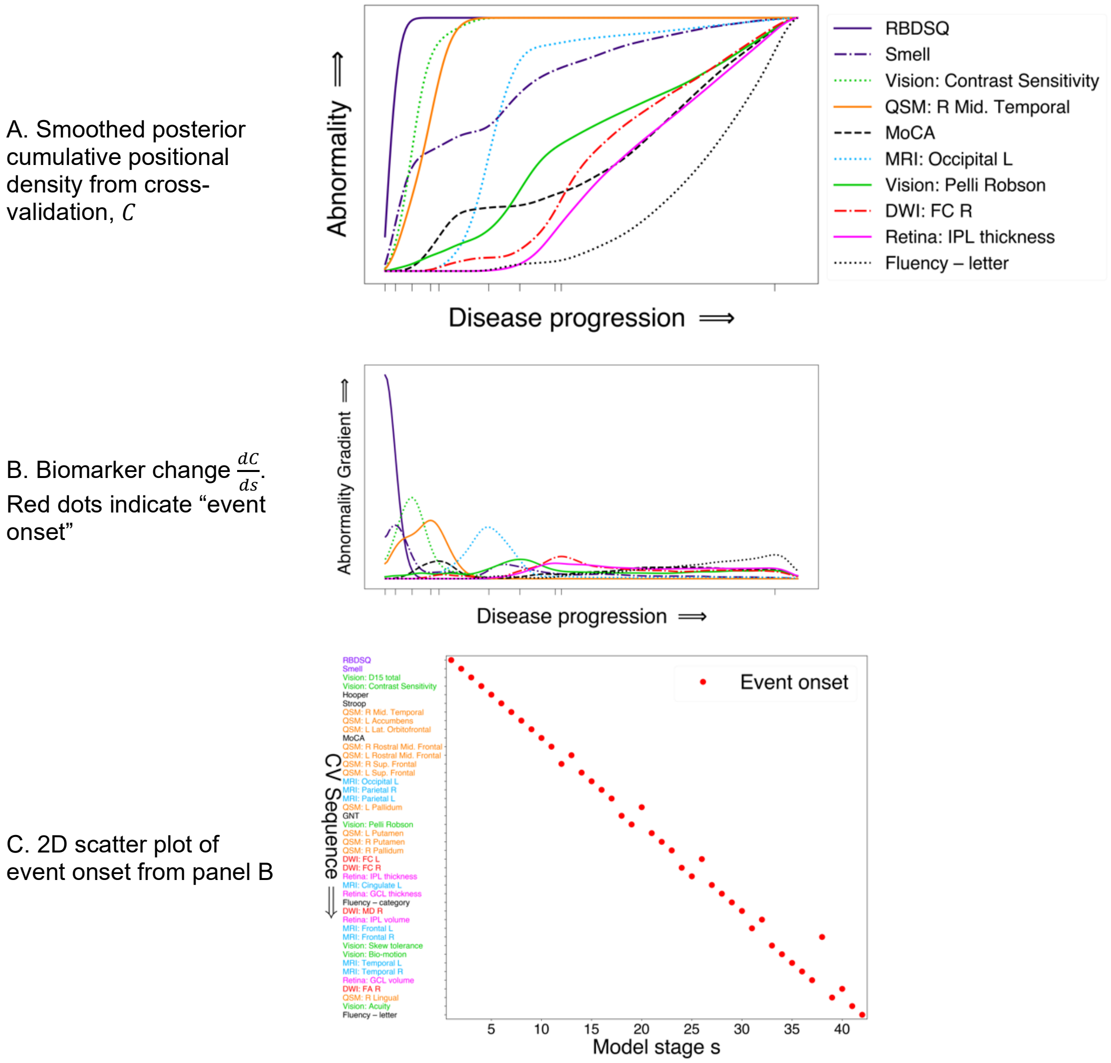

Supplementary Figure 2. ***Cross-validated model sequence estimated using "event onset".*** ***A****: Smoothed posterior cumulative positional density, C.* ***B****: Biomarker change as a function of sequence position s, dC/ds — the red dots show event onset.* ***C****: 2D scatter plot of sequence against event onset.*

3.1.2. Similarity reference: randomised models

As described in the main manuscript, we use the Bhattacharyya coefficient BC as a similarity measure between event-based models across the CV folds. This summarises the statistical overlap of the posterior positional density maps as a single number between zero and one. We calculate BC pairwise between all 50 CV models, then calculate the average similarity of each model (fold) with the other 49. Our overall CV similarity is reported as the mean and standard deviation of these 50 similarity scores (see Supplementary Section 3.2 below).

To provide some context, we calculate a reference distribution for BC by repeating the above procedure on 100 randomisations of the posterior positional density maps. As shown below

in Supplementary Figure 3 (right, inset), this gives a reference similarity score of $BC_0 = 0.37 \pm 0.02$ for randomised posteriors.

3.2. Cross-validation: supplementary results

Supplementary Figure 3 shows CV similarity of our posteriors by event (left panel) and across folds (right panel). Events having high similarity across CV folds showed consistent posterior positional density. Events having lower similarity across folds may be due to heterogeneity in Parkinson's disease progression, e.g., smell-early vs smell-late patients. See Discussion in the main manuscript for planned future work to unravel this heterogeneity.

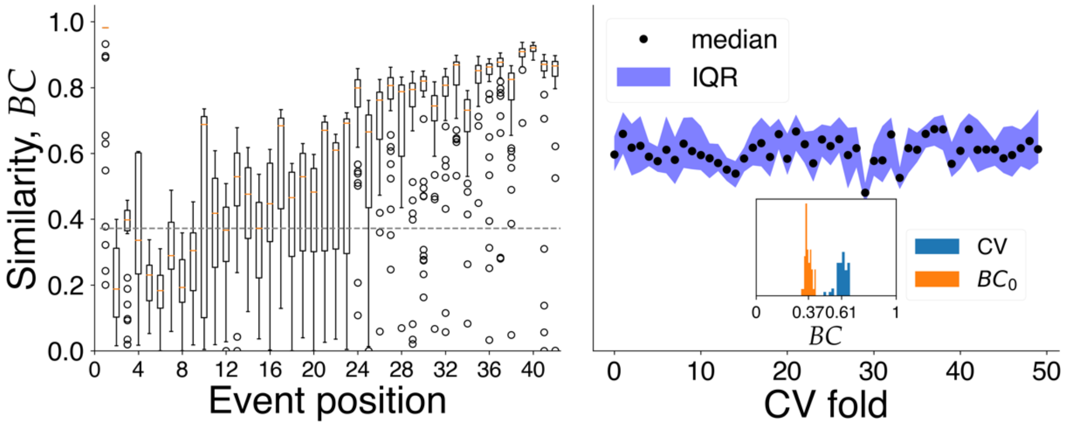

Supplementary Figure 3. ***Cross-validation similarity by event and CV fold (full model).***
Left: BC distributions for each event (each row of the posterior positional density map), across 50 CV models, showing BC_0 (dashed grey line) for reference.
Right: BC distributions for each CV model/fold (the full posterior positional density map) shown as median and interquartile range (IQR). Right, inset: histogram across all folds, including reference BC_0 calculated from random permutations of the posterior positional density maps (see text).

4. Robustness to PDD-HR threshold (age at onset)

The average age at diagnosis of Parkinson's dementia is not known, but the literature suggests that Parkinson's dementia is diagnosed on average 10 years after Parkinson's symptom onset and is most common after age 70 (Lewis *et al.*, 2016; Williams-Gray *et al.*, 2013). Thus, Parkinson's patients are at elevated risk of progressing to Parkinson's dementia if their age at onset (of Parkinson's diagnosis, not dementia) exceeds 60 years old.

To fit our models of progression to Parkinson's dementia, we focussed on high-risk patients ("PDD-HR") whose age at onset was ≥ 65. We verified the robustness of our results by repeating the main experiment using PDD-HR age at onset thresholds of 60 and 70 years, then comparing:

1) the probability of abnormality for each event/feature included in the model — the more this score changes with the threshold, the more likely it is that the event-based model sequence will differ; and
2) the event-based model sequences using Kendall's tau rank correlation.

Supplementary Table 2 shows the effect of the PDD-HR age at onset threshold on the probability of abnormality. We summarise this in a single number by reporting the marker value x for which $p(x|\text{Event}) = 0.5$, as well as the relative ratio x/x_{65} to show the magnitude of the change with respect to the threshold used in our main experiments. We report only those markers affected by the PDD-HR threshold by at least 5%. We report covariate-adjusted values (age, gender, years of education: see main manuscript for details).

We found that increasing the PDD-HR age at onset threshold tended to accentuate the value of x for which $p(x|\mathrm{Event}) = 0.5$, for most markers. That is, the event "occurs" earlier — at a less severe score or biomarker value — because these older patients have more severe pathology and symptoms. For example, QSM markers decrease, and symptoms get worse. This supports the existing notion that older age at onset bestows a higher risk of progressing to dementia. However, this tendency was not universal, which we suspect is due to disease heterogeneity that we hope to unravel in future work.

Supplementary Table 2. Effect of PDD-HR age at onset threshold (60/65/70 years) on marker value x where event probability $p(x|Event) = 0.5$. Differences shown as ratio in parentheses (relative to 65 years threshold). Features not shown displayed differences not exceeding $\pm 5\%$.

**Marker, x**	**Age at onset ≥ 60** PDD-HR n=50	**Age at onset ≥ 65** PDD-HR n=36	**Age at onset ≥ 70** PDD-HR n=18
DWI: FA R	0.479 (0.95)	0.506	0.497 (0.98)
DWI: FC L	–0.032 (1.07)	–0.03	–0.023 (0.75)
Stroop	46.982 (0.84)	55.865	74.556 (1.33)
Vision: Contrast Sensitivity	0.008 (0.13)	0.061	0.07 (1.14)
Vision: Skew Tolerance	0.968 (0.96)	1.004	0.366 (0.36)
Vision: Bio-motion	–5.936 (1)	–5.936	–3.795 (0.64)
Vision: Pelli Robson	1.772 (1)	1.778	1.49 (0.84)
Vision: Acuity	0.138 (0.97)	0.143	0.061 (0.42)
QSM: R Mid. Temporal	0.013 (0.92)	0.014	0.011 (0.77)
QSM: L Lat. Orbitofrontal	0.02 (1.02)	0.019	0.012 (0.62)
QSM: R Rostral Mid. Frontal	0.013 (1.21)	0.01	0.011 (1.03)
QSM: L Rostral Mid. Frontal	0.013 (1.09)	0.012	0.007 (0.62)
QSM: L Sup.Frontal	0.014 (1.03)	0.014	0.008 (0.6)
QSM: R Sup. Frontal	0.012 (0.99)	0.013	0.011 (0.91)
QSM: R Pallidum	0.082 (1.01)	0.081	0.076 (0.94)
QSM: R Putamen	0.053 (0.985)	0.054	0.057 (1.06)
QSM: R Lingual	0.02 (1.158)	0.017	0.013 (0.74)

Supplementary Figure 4 is a scatter plot of event-based model sequences using alternative PDD-HR age at onset thresholds. We found high (and statistically significant) rank correlation of the estimated sequences for age at onset thresholds of 60 (PDD-HR n=50, blue dots) and 70 (PDD-HR n=18, orange stars) with our main model that used a threshold of 65 (PDD-HR n=36). Kendall's tau correlations were $\tau = 0.65$ ($p < 2 \times 10^{-9}$) and $\tau = 0.58$ ($p < 7 \times 10^{-8}$), respectively.

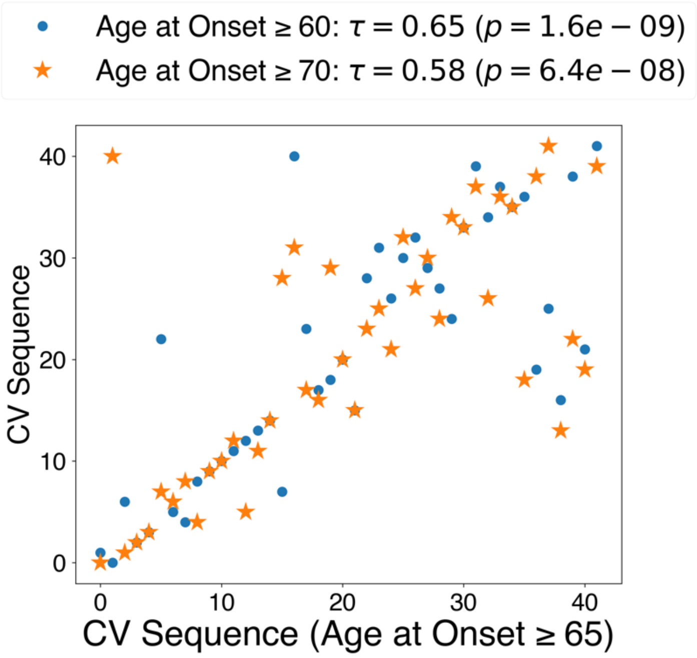

Supplementary Figure 4. ***Effect of age at onset threshold on event-based model sequence of progression to Parkinson's dementia.***
Scatter plot to visualise the correlation between event-based model sequences estimated using a lower (60 years) and higher (70 years) threshold for defining the PDD-HR group (vertical axis) than the base event-based model in the main manuscript (65 years, horizontal axis).

5. Cortical thinning model

Supplementary Figure 5 is a visualisation of the model-based pattern of cumulative abnormality in cortical thickness from Figure 4 (main manuscript), using Brain Painter (Marinescu et al., 2019). Cortical thinning is a late event in our data-driven model of Parkinson's disease progression, with occipital and parietal lobes being the earliest regions affected.

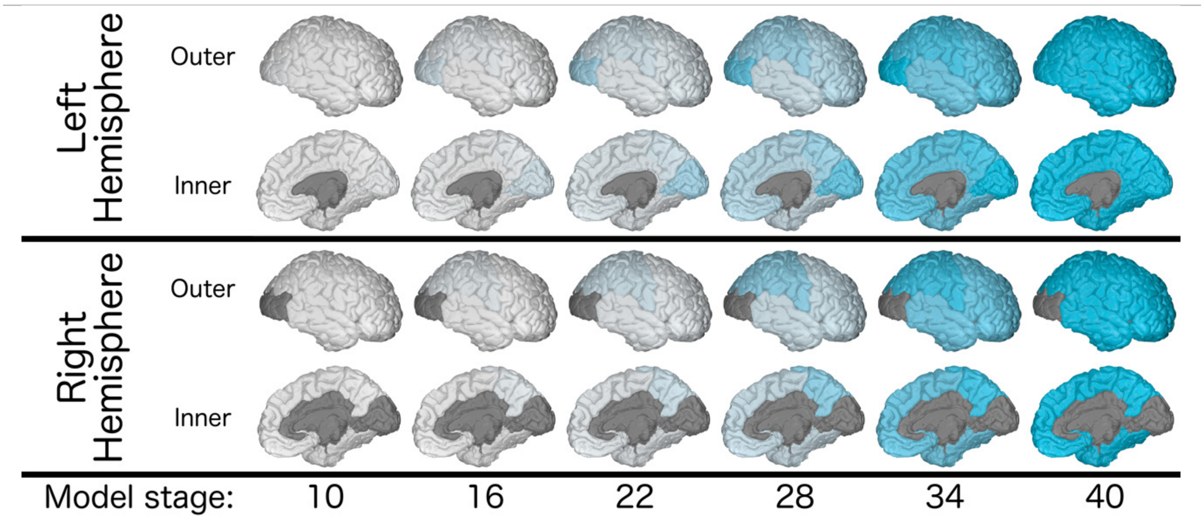

Visual representation of cortical thinning model

Supplementary Figure 5. ***Estimated sequence of cortical thinning (discovery cohort).*** *Visualisation of cortical thinning generated using Brain Painter (Marinescu et al., 2019). Lower: right hemisphere. Upper: left hemisphere (reflected orientation for comparison). Regional cumulative abnormality (0 to 1) is proportional to colour intensity.*

6. Cognitive decline in patients enriched for dementia risk

We analysed cognitive decline on the MoCA in both cohorts. Supplementary Table 3 shows the results of comparing the PDD-HR and PDD-LR groups in each cohort at baseline and followup.

Supplementary Table 3. Cognitive decline in patients (both cohorts).
MoCA scores are shown as median ± median absolute deviation with (mean ± standard deviation) in parentheses.
Abbreviations: MoCA – Montreal Cognitive Assessment; MWU – Mann-Whitney U

MoCA scores	PDD-HR	PDD-LR	MWU test
**VIPD**			
Baseline	**28** ± 1 (27.6 ± 2.2) n=36	**29** ± 1 (28.2 ± 1.7) n=64	u=964 (p=0.084)
First followup (mean 1.3 years)	**27** ± 1.5 (27.1 ± 2.6) n=28	**29** ± 1 (28.1 ± 2.1) n=55	u=593 (p=0.042)
**PPMI**			
Baseline	**27** ± 2 (26.8 ± 2.2) n=146	**28** ± 1 (27.4 ± 2.3) n=206	u = 12590 (p = 0.004)
First followup (mean 1.0 years)	**25** ± 2 (25.2 ± 2.8) n=125	**27** ± 2 (27.0 ± 2.6) n=188	u = 7130 (p < 1e–8)

7. Genetic variation

We investigated dementia-specific genetic variation in both cohorts. We excluded a small number of *GBA*-positive participants (discovery: n=7 patients; external: n=46 patients, n=10 controls), which is a risk factor for more rapid progression to dementia, especially in younger PD patients. We excluded these cases as they are likely to have a more rapid progression to dementia and may show a divergent sequence of events (Blauwendraat *et al.*, 2020). The low number in our cohort precludes a statistical analysis, but we note that our analyses are essentially unchanged when these patients are included: the estimated sequences with/without *GBA* cases (not shown) have high rank correlation with Kendall's tau of $\tau = 0.75$ ($p = 2 \times 10^{-12}$).

For included patients with available genetic information we examined genetic variation due to *MAPT* (H1/H1 haplotype) and *APOE4* (at least one e4 allele), both of which increase risk for dementia. Specifically, we performed Mann-Whitney U tests of group differences in two key Parkinson's disease dementia measures: UPDRS-3 (motor) scores and MoCA scores.

Genetic testing of our patients is approximately 2/3 complete, with the following information currently available for 87 of 141 individuals:

	Carriers (noncarriers)		
**Group** (genetic information available)	**GBA**	**APOE4**	**MAPT** H1/H1
**HC** (n=17 of 34, 50%)	0 (17)	3 (14)	11 (6)

**PDD-HR** (n=23 of 37, 62%)	1 (22)	7 (16)	15 (8)
**PDD-LR** (n=47 of 70, 67%)	6 (41)	9 (38)	33 (14)

The data can support comparisons between genetic subgroups (carriers vs non-carriers) of patients (PDD-HR and PDD-LR combined) for *APOE4* and *MAPT*. The genetic breakdown for both cohorts is as follows:

**PD patients (GBA excluded)**	**Carriers (noncarriers)**	
**Cohort** (genetic information available)	**APOE4**	**MAPT** H1/H1
Discovery cohort (n = 63 of 100)	13 (50)	44 (19)
PPMI cohort (*APOE*: n = 321 of 353) (*MAPT*: n = 349 of 353)	87 (234)	127 (222)

Mann-Whitney U tests of carriers versus non-carriers in UPDRS-3 and MoCA all produce $p \geq 0.19$ (discovery cohort) and $p \geq 0.13$ (PPMI) — see below. From this we conclude that these dementia-specific risk factors do not influence PD progression in our study and that larger numbers may be required to detect differences in sequences between these genetic variations.

		**Carriers**	**Non-carriers**	**MWU test**
**Discovery cohort**				
UPDRS-3 median (MAD)	MAPT	18.5 (5.5)	22 (6)	u=390 (p=0.34)
	APOE4	20 (6)	18.5 (6.5)	u=287 (p=0.26)
MoCA median (MAD)	MAPT	29 (1)	29 (1)	u=361 (p=0.19)
	APOE4	29 (1)	29 (1)	u=323 (p=0.49)
**PPMI cohort**				
UPDRS-3 median (MAD)	MAPT	21 (6)	19.5 (5.5)	u=13696 (p=0.33)
	APOE4	19 (5)	20 (6)	u=9335 (p=0.13)
MoCA median (MAD)	MAPT	28 (1)	28 (1)	u=14056 (p=0.48)
	APOE4	28 (2)	27 (2)	u=9649.5 (p=0.23)

8. Cross-cohort Model Comparison

Supplementary Figure 6 shows comparable event-based model posterior density maps for the discovery and external cohorts. For comparison, individual events have been averaged into modality-based features. For example, all diffusion weighted imaging features of white matter neurodegeneration are averaged into a single row labelled DWI — likewise for cortical neurodegeneration from MRI, and verbal fluency. The ordering (vertical axis) was determined by the median position in the full sequence (Figure 2, main manuscript). The models show high concordance with Kendall's tau rank correlation of $\tau = 0.87$ ($p = 0.017$) and Bhattacharyya coefficient of $BC = 0.96$.

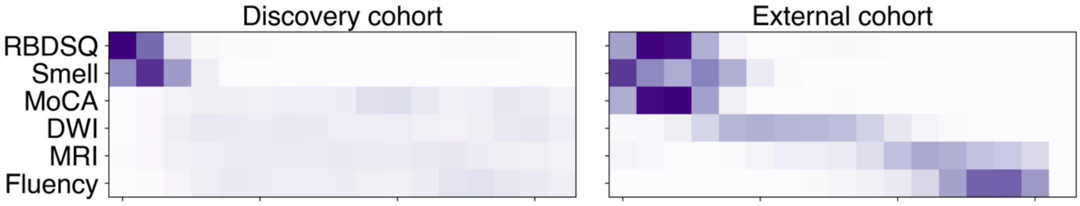

Supplementary Figure 6. ***Cross-cohort comparison.***
Event-based model posterior density maps for discovery cohort (left) and external cohort (right), with imaging events (DWI neurodegeneration in the substantia nigra, MRI cortical thinning) and verbal fluency events averaged together into individual rows.

9. Dysautonomia in Parkinson's progression: SCOPA-Aut

Baseline measures of autonomic function are not available in our discovery cohort but are available in the external data set (PPMI) via the SCOPA-Aut score (Scales for Outcomes in Parkinson's Disease – Autonomic Dysfunction). Supplementary Figure 7 shows the corresponding event-based model with SCOPA-Aut included. The positional density map shows that, as expected, dysautonomia is an early event along with olfactory and sleep abnormalities.

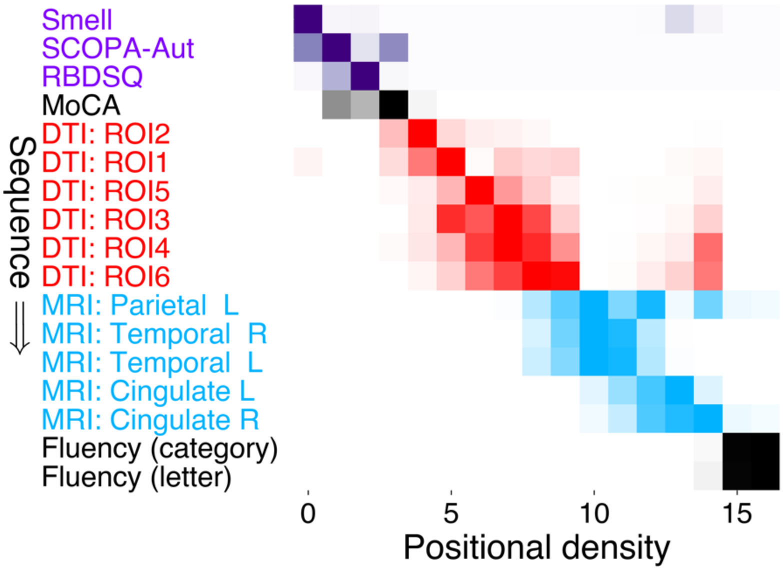

Supplementary Figure 7. ***Event-based model of Parkinson's progression including autonomic function.*** *The data and model estimate that autonomic dysfunction (SCOPA-Aut score) is an early event in Parkinson's disease progression.*

References

Aisen PS, Petersen RC, Donohue MC, Gamst A, Raman R, Thomas RG, et al. Clinical core of the Alzheimer's disease neuroimaging initiative: Progress and plans. Alzheimer's & Dementia 2010; 6: 239–246.

Arrow KJ. A Difficulty in the Concept of Social Welfare. Journal of Political Economy 1950; 58: 328–346.

Blauwendraat C, Reed X, Krohn L, Heilbron K, Bandres-Ciga S, Tan M, et al. Genetic modifiers of risk and age at onset in GBA associated Parkinson's disease and Lewy body dementia. Brain 2020; 143: 234–248.

Firth NC, Primativo S, Brotherhood E, Young AL, Yong KXX, Crutch SJ, et al. Sequences of cognitive decline in typical Alzheimer's disease and posterior cortical atrophy estimated using a novel event-based model of disease progression. Alzheimer's & Dementia 2020; 16: 965–973.

Fonteijn HM, Modat M, Clarkson MJ, Barnes J, Lehmann M, Hobbs NZ, et al. An event-based model for disease progression and its application in familial Alzheimer‘s disease and Huntington’s disease. NeuroImage 2012; 60: 1880–1889.

Frisoni GB, Fox NC, Jack CR, Scheltens P, Thompson PM. The clinical use of structural MRI in Alzheimer disease. Nature Reviews Neurology 2010; 6: 67–77.

Huang J, Alexander D. Probabilistic Event Cascades for Alzheimer's disease. In: Bartlett P, Pereira FCN, Burges CJC, Bottou L, Weinberger KQ, editor(s). Advances in Neural Information Processing Systems 25. 2012. p. 3104–3112.

Jack CR, Knopman DS, Jagust WJ, Shaw LM, Aisen PS, Weiner MW, et al. Hypothetical model of dynamic biomarkers of the Alzheimer's pathological cascade. The Lancet Neurology 2010; 9: 119–128.

Lawton M, Hu MTM, Baig F, Ruffmann C, Barron E, Swallow DMA, et al. Equating scores of the University of Pennsylvania Smell Identification Test and Sniffin‘ Sticks test in patients with Parkinson’s disease. Parkinsonism & Related Disorders 2016; 33: 96–101.

Lewis SJ, Gangadharan S, Padmakumar CP. Parkinson’s disease in the older patient. Clin Med 2016; 16: 376.

Marinescu RV, Eshaghi A, Alexander DC, Golland P. BrainPainter: A Software for the Visualisation of Brain Structures, Biomarkers and Associated Pathological Processes. In: Zhu D, Yan J, Huang H, Shen L, Thompson PM, Westin C-F, et al., editor(s). Cham: Springer International Publishing; 2019. p. 112–120.

Venkatraghavan V, Bron EE, Niessen WJ, Klein S. Disease progression timeline estimation for Alzheimer's disease using discriminative event based modeling. NeuroImage 2019; 186: 518–532.

Williams-Gray CH, Mason SL, Evans JR, Foltynie T, Brayne C, Robbins TW, et al. The CamPaIGN study of Parkinson's disease: 10-year outlook in an incident population-based cohort. J Neurol Neurosurg Psychiatry 2013; 84: 1258.

Young AL, Oxtoby NP, Daga P, Cash DM, Fox NC, Ourselin S, et al. A data-driven model of biomarker changes in sporadic Alzheimer's disease. Brain 2014; 137: 2564–2577.
